# Early use of probiotics might prevent antibiotic-associated diarrhea in elderly (>65 years): a systematic review and meta-analysis

**DOI:** 10.1186/s12877-022-03257-3

**Published:** 2022-07-06

**Authors:** Liying Zhang, Xiaofeng Zeng, Daxin Guo, Yupei Zou, Huatian Gan, Xiaoli Huang

**Affiliations:** 1 The Center of Gerontology and Geriatrics, National Clinical Research Center for Geriatrics, West China Hospital, Sichuan University, Chengdu, Sichuan Province China; 2 Laboratory of Inflammatory bowel disease, the Center for Inflammatory Bowel Disease, Clinical Institute of Inflammation and Immunology, Frontiers Science Center for Disease-related Molecular Network, West China Hospital, Sichuan University, Chengdu, Sichuan Province China

**Keywords:** Probiotics, Antibiotic-associated diarrhea, Elderly, Morbidity

## Abstract

**Background:**

Antibiotic-associated diarrhea (AAD) is diarrhea associated with consuming antibiotics that cannot be explained by other causes. AAD prolongs admission time and increases mortality and financial costs. Elderly individuals are more prone to receive antibiotic treatment and develop AAD. The finding that living probiotic microorganisms decrease AAD incidence in adults (<65 years) has been clarified. However, it is controversial among elderly individuals.

**Methods:**

We aimed to explore whether probiotics could prevent AAD in elderly individuals. We searched three electronic databases (PubMed, EMBASE, and The Cochrane Library), and two reviewers independently screened and assessed the studies. RevMan5.4 software was used to perform a meta-analysis according to the PRISMA guidelines.

**Results:**

Eight RCTs of 4691 participants were included. We excluded two large studies because probiotics were used 48 hours after the first dose of antibiotics, and there was no effect. Subgroup analysis of 6 RCTs showed that probiotics given within two days of antibiotic treatment produced a lower AAD prevalence rate in elderly individuals.

**Conclusion:**

We recommend that elderly individuals could be routinely distributed probiotics to prevent AAD development when receiving antibiotic treatment.

**Trial registration:**

The review was not registered.

**Supplementary Information:**

The online version contains supplementary material available at 10.1186/s12877-022-03257-3.

## Background

Antibiotic-associated diarrhea (AAD) is a side effect of antibiotic consumption symptoms and frequently occurs in inpatients exposed to broad-spectrum antibiotics [1]. The incidence varies according to the type of antibiotics. Antibiotics are classified into different categories according to their risk of leading to AAD [2]. The incidence of diarrhea in adults who receive antibiotic treatment is 5%-70%. In addition,10%-25% of these patients have Clostridium difficile-associated diarrhea (CDAD) [2]. The clinical features caused by Clostridium difficile, which range from uncomplicated diarrhea to pseudomembranous enteritis, are life-threatening [1].

Because of age, comorbidities, intestinal flora changes, frequent hospitalization, and extensive use of antibiotics, elderly individuals are more prone to antibiotic-associated diarrhea [3–5]. The occurrence of AAD prolongs the admission time, increases the economic cost, decreases the quality of life [5, 6], and even increases mortality [5].

Probiotics consist of Streptococcus thermophilus, Enterococcus species, yeast species, and various Lactobacillus and bifidobacteria. Primary and secondary studies have shown that using probiotics during antibiotic administration can decrease the incidence of antibiotic-associated diarrhea [5, 7–9]. However, this conclusion is mainly confirmed in children and nonelderly adults [10, 11]. The effect of probiotics on the prevention and treatment of antibiotic-associated diarrhea in the elderly is [1–5, 12, 13]. Several studies suggest that probiotics may not reduce the risk of AAD or CDAD in older patients [14–16].

A study has hypothesized that probiotics are useless in the elderly's development of AAD because the usage of probiotics is not in time [17]. However, no study exists exploring the first dose time of using probiotics and AAD incidence in elderly individuals. Therefore, we performed the present study.

## Methods

We performed the present review according to the PRISMA (Preferred Reporting Items of Systematic reviews and Meta-Analyses) guidelines [18]. The study was not registered.

### Retrieval strategy

Retrieving the PubMed, Embase, and The Cochrane Library databases, we screened studies concerning probiotics and antibiotic-associated diarrhea in elderly individuals. We limited the article's publication time to May 11, 2021. The retrieval strategy is listed in Appendix 1.

### Eligibility and exclusion criteria

Inclusion criteria**:** (1) research subjects: age ≥65 and receiving antibiotic treatment for any reason; (2) intervention: any kind or dose of probiotics; (3) outcome indicators: the incidence of antibiotic-associated diarrhea; and (4) randomized controlled trials. Exclusion criteria: (1) not RCT; (2) review, conference proceedings, literature on animal experiments; (3) duplicate reports; (4) incomplete data or unacquirable literature;

### Study selection and data extraction

Two evaluators (LYZ and XFZ) screened the documents and extracted information independently. If there were disagreements, they consulted a third party (XLH) to resolve the difference. First, we screened the title and abstract to exclude irrelevant literature. They then screened the whole text carefully. The contents of data extraction included (1) Basic information, such as the title, the first author, publication time, and region. (2) Basic characteristics of subjects: the number of participants, age, and sex distribution in each group; (3) The key elements of the bias assessment; (4) The definition of AAD, the number and incidence of the participants with antibiotic-associated diarrhea.

### Quality assessment

Two reviewers (LYZ and XFZ) independently evaluated the risk of bias in the studies using the risk of bias 2(ROB2) tool for randomized studies. Then they cross-check the evaluation scores results. When there were disagreements, they consulted the third reviewer (XLH) to resolve the differences. Since there were <10 included studies, we did not apply a funnel plot to observe publication bias.

### Data analysis

The meta-analysis progression (qualitative synthesis) was carried out using RevMan5.4 software, using the standardized mean difference (SMD) as the measurement data-effect indicator and supplying the point estimate and 95% CI. The heterogeneity among the studies was evaluated by the χ2 test (test level is α=0.1). The I^2^ was used to judge the heterogeneity quantitatively. I^2^ values greater than 25% were considered low heterogeneity, 50% moderate, and 75% high heterogeneity. We used the fixed-effects model and inverse variance method to evaluate the data if I^2^<50%; otherwise, we executed the random effects model and the DS-L method for data analysis. When the synthetic analysis is unavailable, a descriptive statement will be provided. When significant heterogeneity existed, we performed subgroup analysis to carry out further studies. We performed sensitivity analysis by reducing some included studies.

## Results

### Study selection

**Fig. 1:**
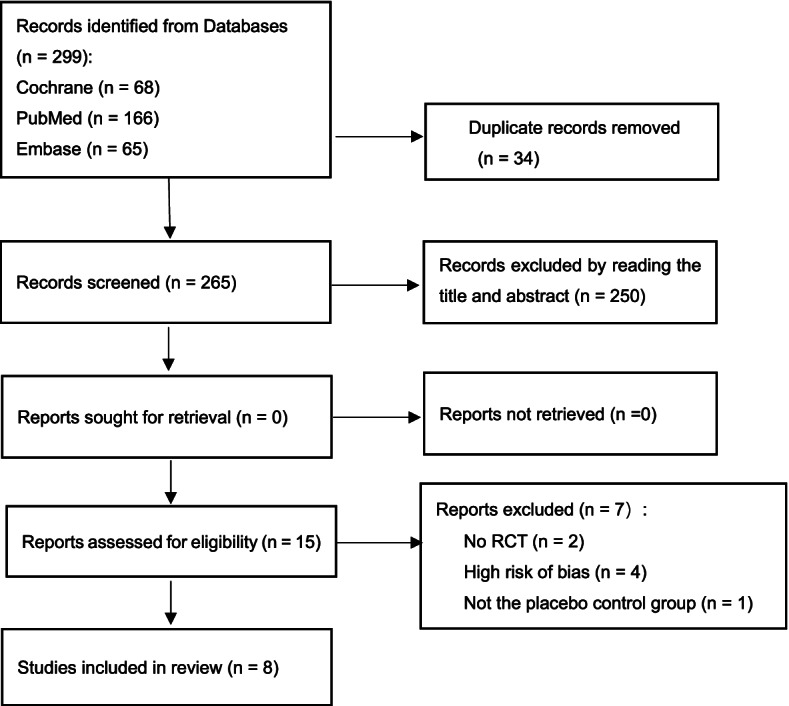
The study selection process

The details of the study selection are shown in Fig 1. A total of 299 pieces of literature were selected from three databases. Thirty-four records were removed because of duplication. Two hundred fifty irrelevant studies were excluded after screening the titles and abstracts. Seven records were excluded after we screened the full text, and four records were excluded because of high risks of bias. Two records were not RCTs, and one was excluded because of the 'probiotics' control group (the participants in the control group were dispensed partial probiotics). Finally, eight pieces of literature [1–5, 19–21] from 4689 participants were selected for the meta-analysis.

### Study characteristics

Four of 8 studies were conducted in England. Almost all of the participants were in the hospital when they were enrolled. The patients in the control group were all given the placebo. Meanwhile, the patients in the experimental group who received the probiotics varied in species, dosage, and course. A total of 624 participants in 6 studies accepted the probiotics within two days of the first dose of the antibiotic treatment. The prevalence of AAD varied from 10.1% to 21.21% in the probiotics group and from 8.70% to 35.56% in the placebo group. The details are shown in Table 1.

**Table 1:** The characteristics of the studies

Author, years	country	setting	Type of probiotics	Time from antibiotic to probiotic	Follow-up time	Probiotics group (n)	Placebo group (n)	AAD in Probiotics group (n)	AAD in Placebo group (n)
Lewis, 1998 [5]	England	Hospital	Saccharomyees boulardii	Within 48 h	No report	33	36	7	5
Beausoleil, 2007 [19]	Canada	Hospital	Lactobacillus acidophilus and Lactobacillus casei.	Within 48 h	for 21 days after the last dose of antibiotic	44	45	7	16
Hickson, 2007 [20]	England	Hospital	Lactobacillus casei, S thermophilus, and L bulgaricus	Within 48 h	Antibiotics treatment plus 28days, or for 28 days from discharge	69	66	7	19
Safdar, 2008 [21]	America	Hospital	Lactobacillus acidophilus.	Within 24 h	Unspecified (probably for 14 days after the last dose of antibiotic)	23	17	4	6
Pozzoni, 2012 [2]	Italy	Hospital	S. boulardii	Within 48 h	for 12 weeks after the last dose of antibiotic	106	98	16	13
Allen, 2013 [1]	England	Hospital	Lactobacillus acidophilus and bifidobacterium	Within 7days	for 8 weeks after recruitment	1470	1471	159	153
Wright, 2015 [4]	Australia	Hospital	Lactobacillus casei and Shirota strain	Within 24 h	Unspecified (probably 28 days)	41	46	5	4
C. Rajkumar, 2020 [3]	England	Hospital	L. casei DN114001, L. delbrueckii subspecies bulgaricus, and S. thermophilus	Within 7days	for two weeks after the last dose of antibiotic	549	577	106	103

### AAD assessment

The studies were carried out from 1998 to 2020. The definition of diarrhea was different from study to study. One study [1] defined diarrhea as three or more loose stools in a 24 h period or as stools described as looser than daily, and the follow-up time was eight weeks after the participants enrolled in the group. While Mary Hickson et al. [20] defined diarrhea as more than two liquid stools a day for three or more days in quantities over regular. The participants were followed-up until one week after the antibiotic treatment. The incidence of AAD was higher in the probiotics group, as shown in 5 studies [1–5] involving 4427 objects. In addition, 4067 of 4691 patients received probiotics not in a timely manner (2 days after the first dose of the antibiotic). Six hundred seventy-six patients in 6 studies [2, 4, 5, 19–21] received probiotics within two days from antibiotic to probiotic, showing a significant reduction in AAD incidence in the probiotics group.

### Risk of bias in studies

**Fig 2.**
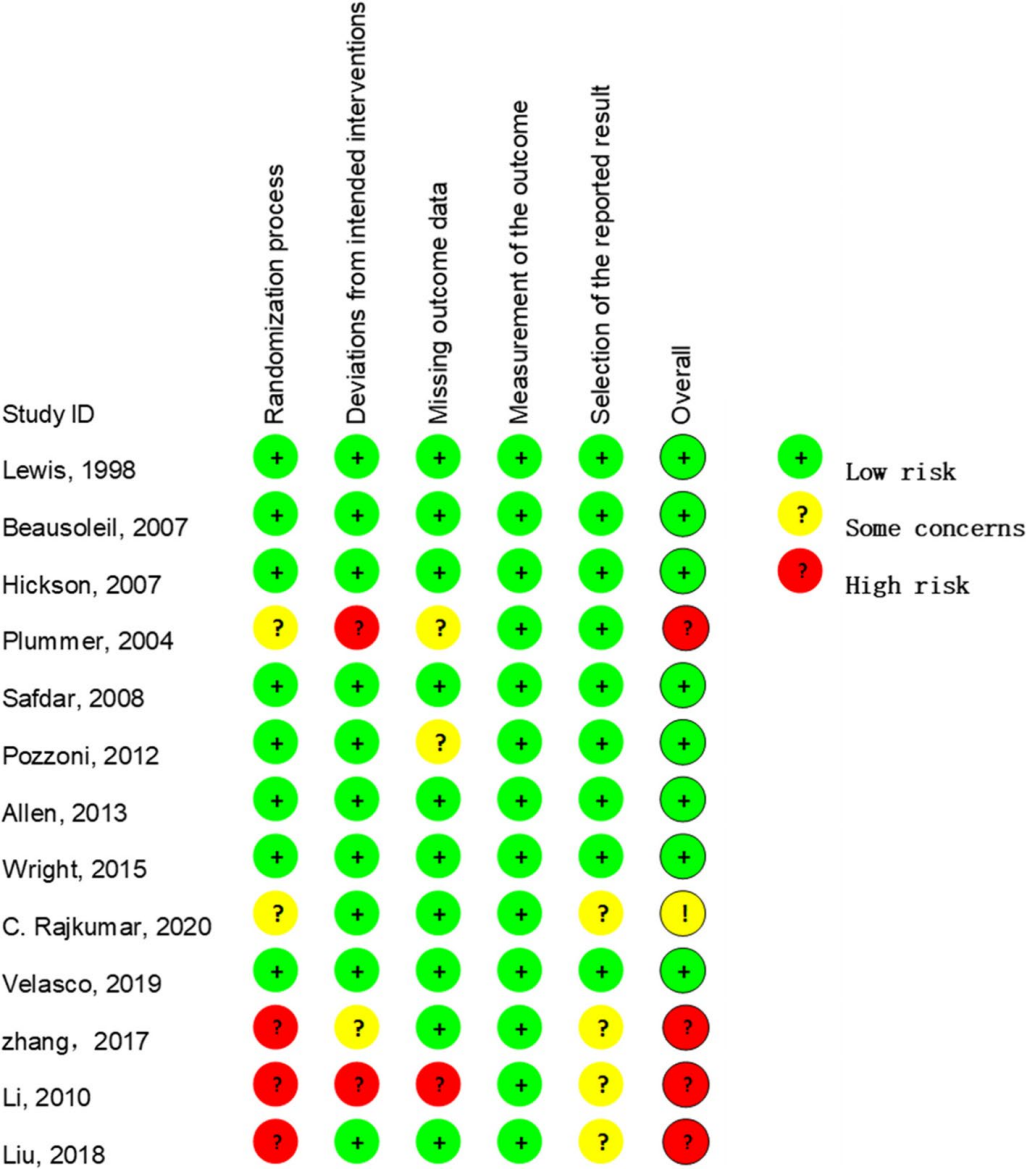
The risks of bias of the 13 studies

We employed the Risk Of Bias 2 (ROB2) to assess the risk of bias in all studies. Seven of the 8 RCTs had a low risk of bias. One was evaluated as some concern. The details of the assessment results of the included studies are shown in Fig 2.

### Results of syntheses

**Fig 3.**
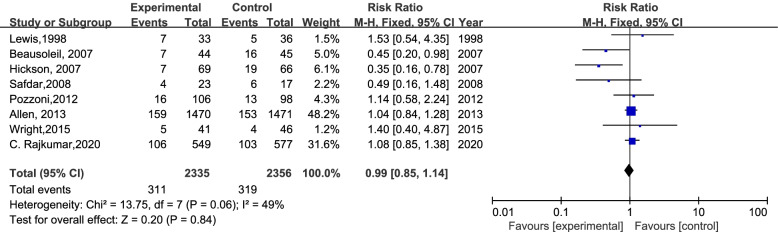
The forest plot of the included studies

**Fig 4.**
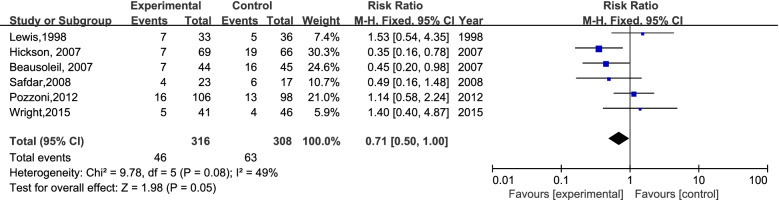
The forest plot of the six studies (within 48 h)

**Fig 5.**
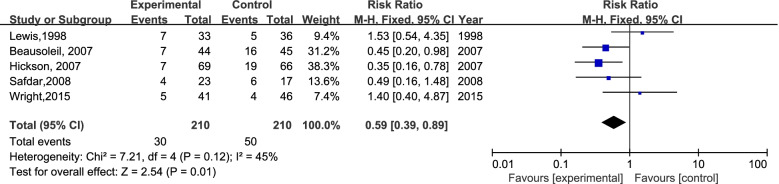
The forest plot of the five studies

**Fig 6.**
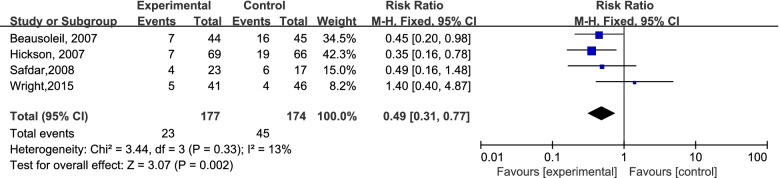
Sensitivity analysis (excluding the earliest study)

A total of 4691 participants from 8 RCTs were involved; 630 patients experienced antibiotic-associated diarrhea, and 4061 patients did not have AAD. The incidence of AAD was 13.32% (311/2335) in the probiotics group and 13.54% (319/2356) in the placebo group. In the case of I^2^ < 50%, we chose the fixed-effects model to perform the meta-analysis. There was no significant difference in the incidence of AAD between the probiotic group and placebo group (RR=0.99; 95% CI,0.85-1.14; P=0.84; I^2^=49%). The details are shown in Fig 3. Previous secondary research has observed moderate heterogeneity (I^2^: 25%-50%). Eight studies were divided into two groups according to the time of first dose of probiotics used (within 48 hours of the first dose of antibiotics or later). Surprisingly, six studies concluded that it is efficient to prevent AAD by using probiotics within 48 h (RR=0.71; 95% CI, 0.71-1.00; P=0.05; I^2^=49%), the details are shown in Fig 4. We excluded the study by Pozzoni. et al. ('missing data bias' is some concerns) [2], and observed the same results: RR=0.59 (95% CI,0.39-0.89; P=0.01; I^2^=45%) (Fig 5). There was no significant difference when probiotics were used later. (RR=1.06, 95% CI, 0.90-1.24; P=0.50; I^2^=0 %). In addition, when we excluded the earliest studies [5] of 6 RCTs to test the heterogeneity, the meta-analysis result was not quantitatively changed (Fig 6) (RR: 0.49, 95% CI: 0.31-0.77; P=0.002; I^2^=13%).

### Quality of the evidence

**Fig 7.**
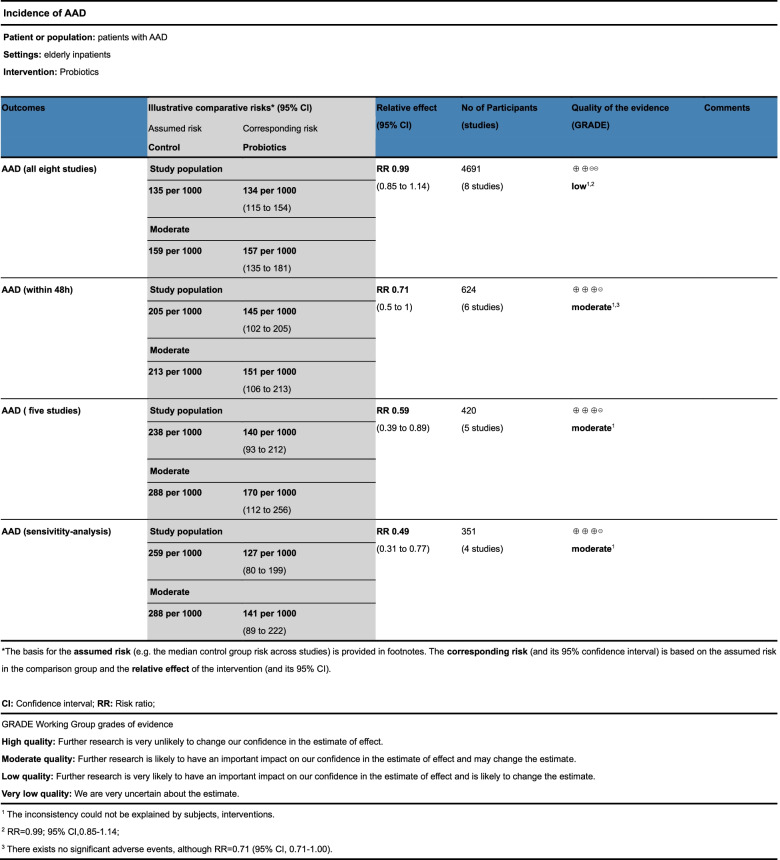
Quality of the evidence

We used the GRADE profiler to evaluate the results of the meta-analysis (Fig 7). The results showed that our outcomes were of moderate quality. Since we included RCT studies, we downgraded the outcomes to moderate quality due to comparatively larger effects.

## Discussion

Presently, AAD is the change in stool number and trait occurring after using an antibiotic. In addition, the definition is not explicit. Some researchers believe that diarrhea occurring within 12 weeks after using the first dose of antibiotic treatment [1, 2] could be defined as AAD. Others were considered within eight weeks [4, 22]. The mechanism of antibiotic-associated diarrhea is also unclear. The widely accepted mechanisms [3, 4, 13] are as follows: (1) Antibiotics destroy diversity and decrease the quantity of intestinal flora. Therefore, exogenous pathogenic bacteria colonize and grow in the intestine. (2) Undigested carbohydrate molecules accumulate in the intestine, leading to osmotic diarrhea. How probiotics prevent AAD development is as follows [1]: (1) Probiotics change the original intestinal pH, which is unsuitable for pathogen reproduction. (2) Probiotics inhibit the secretion of bacterial toxins. (3) Probiotics effectively compete for nutrients and bind sites with pathogenic bacteria. (4) Protecting the immune barrier and the intestinal mucosa. Because of probiotic intolerance, some participants experienced abdominal distension, nausea, and vomiting [12, 17]. Nevertheless, no severe adverse reactions have occurred [2, 5, 17, 20, 23–25], and probiotics are safe in preventing AAD at any age. We explored the association between the time of using the first dose of probiotics and the incidence of AAD in elderly individuals, which is the innovation of a recent study. We conclude that probiotics reduce AAD incidence in the elderly, as first observed.

The study's limitations are as follows: First, only one outcome (AAD incidence) is listed in the present study, which is not comprehensive. Second, only C. Rajkumar et al.'s [3] study involved outpatients living in the nursing home or personal homes, which may limit the applicability of the conclusions of this study. Despite rigorously screening and evaluating the articles to conclude, the conclusion derives from a small number of RCTs of a limited sample size. More large-scale RCT studies designed for elderly individuals related to probiotics and AAD are needed to make the conclusion more robust. Moderate heterogeneity may be attributed to the unclear definition of AAD. We urgently need a clear definition of AAD and follow-up time in the future. In summary, we recommend that elderly individuals routinely distribute probiotics to prevent AAD development when receiving antibiotic treatment.

## Conclusion

In the present study, we performed a subgroup analysis to explore the association of the first dose time of probiotics and AAD incidence in elderly individuals. Although the risk of bias scores of the study conducted by Allen and Rajkumar are acceptable, we excluded these two studies because probiotics were used 48 hours after the first dose of antibiotics in these studies. The subgroup analysis of six RCTs showed that probiotics given within 48 hours of antibiotic treatment produced a lower AAD prevalence rate in elderly individuals, which was proposed in the systematic review and meta-analysis first. It is remarkable that we obtained a more positive result when several studies were excluded step by step (Figs 4, 5, 6). Furthermore, we hold the opinion that the possible reasons why our exclusion of studies affects the outcomes to be positive are as follows: (1) The preventive function of probiotics for AAD is closely associated with the time the probiotic treatment was started, and the function could diminish when probiotics were used later in the elderly inpatients (Fig. 4). Similar claims have been proven in an adult study [26]. (2) A longer probiotic treatment duration results in better prevention of AAD. There was no effect of probiotics on preventing AAD in these studies (4, 5) when probiotics were given only during antibiotic treatment (Fig. 5). (3) There have been many changes in antibiotic stewardship policies in many healthcare systems, which may affect the incidence of AAD in recent years [3]. An adequate follow-up period is essential to observe AAD occurrence [4, 19] (Fig. 6). Because of various AAD definitions, probiotic and antibiotic types, and the follow-up time, the credibility of the conclusion may be affected. A larger scale of studies with a coincident definition of AAD and follow-up time are needed to strengthen the conclusion.

## Supplementary Information

**Additional file 1.**

## Data Availability

All data generated or analysed during this study are included in this published article [1–5, 19–21], and Table 1 The studies' characteristics.

## Abbreviations

AAD: antibiotic-associated diarrhea
RR: relative risk
RCT: random clinical trial
