# Supplementary material for: Early use of probiotics might prevent antibiotic-associated diarrhea in elderly (>65 years): a systematic review and meta-analysis
Source: BMC Geriatr. 2022 Jul 6;22:562. doi: 10.1186/s12877-022-03257-3 (PMC9260993; doi:10.1186/s12877-022-03257-3)
Supplement: Supplementary file 1 — Additional file 1. [file 12877_2022_3257_MOESM1_ESM.docx]

Appendix 1

Cochrane Library

ID Search Hits Result

#1 MeSH descriptor: [Probiotics] explode all trees 2100

#2 MeSH descriptor: [Synbiotics] explode all trees 154

#3 (bifidobacteri*):ti,ab,kw (Word variations have been searched) 3125

#4 (probiotic*):ti,ab,kw (Word variations have been searched) 7330

#5 (synbiotic*):ti,ab,kw (Word variations have been searched) 780

#6 (lactobacill*):ti,ab,kw (Word variations have been searched) 5403

#7 (saccharomyces):ti,ab,kw (Word variations have been searched) 517

#8 #1 OR #2 OR #3 OR #4 OR #5 OR #6 OR #7 10351

#9 (antibiotic associated diarrhea):ti,ab,kw (Word variations

have been searched) 898

#10 #8 AND #9 350

#11 MeSH descriptor: [Aged] explode all trees 211221

#12 (elderly):ti,ab,kw (Word variations have been searched) 51876

#13 #11 OR # 12 645254

#14 #10 AND # 13 68

Embase

No.Query Results Result

#1. 'probiotic agent'/exp OR 'synbiotic agent'/exp OR 158,940

probiotic*:ab,ti OR synbiotic*:ab,ti OR

bifidobacteri*:ab,ti OR lactobacill*:ab,ti OR

saccharomyces: ab,ti

#2. 'antibiotic associated diarrhea'/exp 783

#3. #1 AND #2 470

#4. #3 AND 'randomized controlled trial'/de 65

PubMed

ID Search Hits Results

#1. ((("Probiotics"[Mesh]) OR ((((((((probiotic) OR (bifidobacterial)) OR

(bifidobacterium)) OR (Lactobacillaceae)) OR (lactobacilli)) OR

(lactobacillus)) OR (saccharomyces)) OR (synbiotic))) 209775

#2. (("Anti-Bacterial Agents"[Mesh]) OR

(((((((((((((((((((((((((((((((((Agents, Anti-Bacterial) OR

(Anti Bacterial Agents)) OR (Antibacterial Agents)) OR

(Agents, Antibacterial)) OR (Antibacterial Agent)) OR

(Agent, Antibacterial)) OR (Anti-Bacterial Compounds)) OR (

Anti Bacterial Compounds)) OR (Compounds, Anti-Bacterial)) OR

(Anti-Bacterial Agent)) OR (Agent, Anti-Bacterial)) OR

(Anti Bacterial Agent)) OR (Anti-Bacterial Compound)) OR

(Anti Bacterial Compound)) OR (Compound, Anti-Bacterial)) OR

(Bacteriocidal Agents)) OR (Agents, Bacteriocidal))OR

(Bacteriocidal Agent)) OR (Agent, Bacteriocidal)) OR

(Bacteriocide)) OR (Bacteriocides)) OR

(Anti-Mycobacterial Agents)) OR (Agents, Anti-Mycobacterial)) OR

(Anti Mycobacterial Agents)) OR (Anti-Mycobacterial Agent)) OR

(Agent, Anti-Mycobacterial)) OR (Anti Mycobacterial Agent)) OR

(Antimycobacterial Agent)) OR (Agent, Antimycobacterial)) OR

(Antimycobacterial Agents)) OR (Agents, Antimycobacterial)) OR

(Antibiotics)) OR (Antibiotic)))) 987818

#3. (("Diarrhea"[Mesh]) OR ((Diarrhea) OR (Diarrhoea))) 124673

#4. #1 AND #2 AND #3 1386

#5. Filters: Randomized Controlled
